# Assessing the anesthetic effectiveness of remimazolam in MELAS patients requires careful investigations

**DOI:** 10.1186/s40981-022-00536-1

**Published:** 2022-06-21

**Authors:** Josef Finsterer

**Affiliations:** Neurology & Neurophysiology Center, Postfach 20, 1180 Vienna, Austria

**Keywords:** COVID-19, SARS-CoV-2, Neuroimmunology, Anxiety, Depression

Letter to the Editor

We read with interest the article by Kitaura et al. about a 47-year-old male with mitochondrial encephalopathy, lactic acidosis, and stroke-like episode (MELAS) syndrome, who underwent elective transcatheter mitral valve repair because of mitral insufficiency [1]. MELAS was due to the common variant m.3243A>G and manifested phenotypically with short stature, cerebral and cerebellar atrophy, deafness, hypertrophic cardiomyopathy, Wolff-Parkinson-White syndrome, heart failure, renal insufficiency requiring hemodialysis, myopathy, and lactic acidosis [1]. General anesthesia for the procedure was successfully induced and maintained with remimazolam and remifentanil, without circulatory compromise or metabolic acidosis [1]. It was concluded that remimazolam may be a new anesthetic option for MELAS [1]. The study is attractive but raises concerns that should be discussed.

We do not agree with the statement in the discussion that serum lactate is elevated in MELAS because of metabolic acidosis [1]. It is the other way around. Metabolic acidosis develops because of lactic acidosis, and lactate is produced in tissues with impaired oxidative phosphorylation [2].

The two statements “propofol can be safely used in patients with MELAS” and “it is safer to avoid the continuous administration of propofol in patients with mitochondrial disorders” are contradictory [1]. This discrepancy should be solved. The topic is conflicting as there are reports describing the safe use of propofol but also reports that describe propofol infusion syndrome after propofol administration in mitochondrial disorders [3].

A shortcoming of the study is that the heteroplasmy rate (relation of mutated mtDNA to wild-type mtDNA in a single mitochondrion or single cell) of the m.3243A>G variant was not provided [1]. Knowing heteroplasmy rates in various tissues is crucial as they determine the clinical course and outcome of MELAS patients [4]. It would be also interesting to know the mtDNA copy number (absolute number of mtDNA copies within a mitochondrion or single cell) as it is a further factor determining the phenotype.

We do not agree with the conclusions that remimazolam can be a new option for anesthetizing MELAS patients [1]. Findings in a single patient cannot be generalized. To assess if remimazolam is truly a novel option for anesthetizing MELAS patients, further, appropriately designed studies are warranted.

We should be told what the authors mean with “bilateral pathologic reflex positive” [1]. Do they mean that the tendon reflexes were exaggerated? If this is the case, it would contradict with the statement that there was “weakness of tendon reflexes” [1]. We should be told if there was a mixture of exaggerated and reduced tendon reflexes. Obviously, the index patient had myopathy, implying that tendon reflexes are reduced. However, the patient also had cerebral atrophy, which does not exclude that there was affection of the pyramidal tract.

Missing are the reference limits in the text and Table 1, which makes it impossible to interpret the provided results. Missing is the current medication the patient was regularly taking prior to MitraClip® implantation. Missing is also the previous history, particularly if the patient had generalized anesthesia before and if any complications had occurred.

Overall, the interesting study has some limitations that call the results and their interpretation into question. Clarifying these weaknesses would strengthen the conclusions and could improve the study. Whether remimazolam is beneficial for MELAS in general remains unsupported.
